# Renal surgery for kidney cancer in Germany 2005–2006: length of stay, risk of postoperative complications and in-hospital death

**DOI:** 10.1186/1471-2490-14-74

**Published:** 2014-09-12

**Authors:** Andreas Stang, Christian Büchel

**Affiliations:** 1 Institut für Medizinische Informatik, Biometrie, und Epidemiologie (IMIBE), Universitätsklinikum Halle, Halle, Essen 45147, Germany; 2 School of Public Health, Department of Epidemiology, Boston University, 715 Albany Street, Talbot Building, Boston, MA 02118, USA

**Keywords:** Hospital mortality, Intraoperative complications, Kidney cancer, Length of stay, Nephrectomy, Postoperative complications

## Abstract

**Background:**

Representative statistics of surgical care among patients with kidney cancer are scant. With the introduction of the diagnosis related group system in Germany, it is now possible to provide nationwide statistics on surgical care. We studied in-hospital mortality risk in relation to comorbidity and complications, length of hospital stay in relation to surgical approach and comorbidity, and risk of complications in relation to surgical approach among kidney cancer patients undergoing nephrectomy.

**Methods:**

We analyzed the nationwide hospitalization file of the years 2005 and 2006 including 23,753 hospitalizations with a diagnosis of renal cancer and partial or complete nephrectomy and classified comorbidity (Charlson comorbidity index) and complications. Length of stay, risk of in-hospital complications and in-hospital death were analyzed by linear regression and log-linear regression (relative risks (RR) and 95% confidence intervals (95% CI)).

**Results:**

The overall in-hospital mortality was 1.4%. Per one unit increase of the Charlson comorbidity index, the adjusted risk of in-hospital mortality increased by 53% (95% CI 47-59%). The risks of bleeding or acute posthaemorrhagic anemia, respiratory, urological and gastrointestinal complications and infections ranged between 1.1% and 2.7% with the exception of bleeding or acute posthaemorrhagic anemia with 18.4%. Complications were associated with an increased adjusted in-hospital mortality risk. Highest adjusted mortality risk ratios were observed for gastrointestinal (RR = 3.61, 95% CI 2.32-5.63) and urological complications (RR = 3.62, 95% CI 2.62-5.00). The risk of haemorrhage or acute posthaemorrhagic anemia was lower for total laparoscopic nephrectomies than total open nephrectomies. The adjusted risk of gastrointestinal complications was lower for partial open compared to total open nephrectomy (adjusted RR = 0.66, 95% CI 0.45-0.97). Total laparoscopic nephrectomy was associated with shorter length of stay (−3.3 days; 95% CI 2.9-3.7 days) compared to total open nephrectomy. The estimated age-adjusted increase of length of stay per one unit increase of the Charlson comorbidity index was 1.3 days (95% CI 1.2-1.4 days).

**Conclusions:**

In this representative population-based analysis, we found that the surgical approach is associated with the risk of complications and length of hospital stay. Furthermore, in the era of ageing populations, renal cancer patients with comorbidities should be counseled about their increased in-hospital mortality risk.

## Background

The estimated number of newly diagnosed patients with renal cancer and deaths due to renal cancer is 115,200 cases and 49,000 cases in Europe in 2012, respectively. Renal cancer accounts for 3.3% of all newly diagnosed cancers in Europe (without non-melanoma skin cancer) [1]. Surgical excision is the primary treatment [2]. Since 2006, laparoscopic radical nephrectomy has become an established standard for the surgical treatment of renal cancer [3]. According to the European guidelines, nephron-sparing surgery (partial nephrectomy), is recommended for T1 tumors (≤7 cm diameter) and is an option for T2 tumors [4]. Alternative treatment options include several percutaneous or open ablation approaches. However, the recommended indications for these approaches are small [4].

In 2002, Martin et al. developed 10 criteria for the reporting of surgical outcomes [5]. Its limitation included the lack of procedure-related variables. In 2007, procedure-related complications of nephrectomy have been added [6]. Typical complications after partial or total nephrectomy include pneumothorax, haemorrhage and acute posthaemorrhagic anemia, acute renal failure or insufficiency, postoperative infection including peritonitis and wound infection, and intestinal obstruction [7,8].

Our purpose was to describe the nationwide surgical management of renal cancer in Germany and its patient risk-factors and comorbidities adjusted association with length of stay, complications and in-hospital mortality.

## Methods

In 2004, the DRG (diagnosis related group) reimbursement system became compulsory for hospitals. All hospitals that are recompensated by the DRG-system annually transfer their individual hospitalisation data to the DRG data center (InEK). Hospitals have a strong incentive to report their complete hospitalisation data. The German DRG statistics is virtually a complete collection of hospitalizations all over Germany with a few exceptions. The DRG data center undertakes plausibility checks of the data and generates a plausibility protocol that is sent back to the hospitals. Hospitals can re-submit their corrected data files. Thereafter, the DRG data center forwards anonymised data to the Federal Bureau of Statistics. We were able to use the hospitalization years 2005 and 2006 including overall 36.3 million hospitalizations.

The Federal Law on Statistics (Bundesstatistikgesetz, BstatG) allows the use of DRG hospitalization data for scientific purposes (§ 16, (6), (7)) without ethical review. These data are not publicly available but can be accessed after permission. We received permission from the Federal Bureau of Statistics to access this data.

For each hospitalization, one main diagnosis and up to 99 secondary diagnoses coded can be documented. In 2005, diagnoses were coded according to the ICD-10-GM version of 2005 [9]. In 2006, the ICD-10-GM version 2006 was used [10]. The diagnosis that led to the hospitalization assessed at the end of the hospitalization is defined as the main diagnosis. Up to 100 medical procedures can be coded according to German classification of operations and procedures (OPS), a classification that represents a German version of the International Classification of Procedures in Medicine (ICPM). In 2005 and 2006, the OPS versions of the years 2005 and 2006 respectively were used [11,12].

We retrieved all hospitalizations that included a main diagnosis of renal cancer (ICD-10: C64) and a procedure code for partial, simple, radical and other types of nephrectomy (Additional file 1: Table S1). For the analysis, we only distinguished between partial and total (simple or radical) nephrectomy.

We excluded hospitalizations when 1) renal cancer was only a secondary diagnosis, 2) renal cancer (primary diagnosis) and pelvis cancer (secondary diagnosis) were simultaneously coded, 3) the coding of the surgical approach was ambiguous, or 4) age, gender or places of residence were missing. Based on the type and surgical approach we categorized nephrectomies as total open, total laparoscopic, partial nephrectomies. A small proportion of nephrectomies had unspecific, unclear or implausible coding (Additional file 1: Table S2).

We used a coding algorithm for defining comorbidities in ICD-10 administrative databases [13] to derive the Charlson comorbidity index (CCI) [14]. The German hospitalization file contains up to 100 ICD-10 coded diagnoses. We used these codes to derive the CCI that contains overall 17 chronic diseases with different weighting factors (Additional file 1: Table S3). As all included patients had a renal cancer diagnosis, we removed this item from the calculation of the CCI.

We used an adaption of an established algorithm to derive procedure-related in-hospital complications after nephrectomy [8]. For bleeding, we also derived procedure codes that allowed the identification of bleedings that required blood transfusion (Additional file 1: Table S4). We also extracted age at hospital admission, gender, length of hospital stay in days (LOS) and in-hospital mortality.

### Statistical methods

The unit of analysis was the hospital admission with a diagnosis of renal cancer and a partial or total nephrectomy. We excluded 199 and 209 hospitalizations because surgical coding was arbitrary and demographic data (age, gender, place of residence) was missing respectively. The final data set included 23,753 hospitalizations. For the study of the association between type of nephrectomy and LOS, we used a linear regression with LOS as the outcome variable. We used log linear regression models to estimate relative risks (RR) and 95% confidence intervals (95% CI) for the association between type of surgery (reference group: total open nephrectomy) and dichotomous outcomes like bleeding, bleeding requiring blood transfusion, and in-hospital mortality. We checked the model assumptions by visual inspection of residual plots. Model assumptions were fulfilled. As age and comorbidity are confounders of these associations, we adjusted our analyses for both variables.

## Results

From 2005 through 2006, the overall number of 23,753 nephrectomies included 10.6% renal cancers that either had lymph node or distant metastasis. The median LOS was 11 days and the in-hospital mortality was 1.4%. The sex-specific comparison revealed that women were one year older than men, their median LOS was one day longer than for men, and their median Charlson comorbidity score was zero as opposed to one among men. The probability of partial nephrectomy was lower among women than men (RR = 0.79, 95% CI 0.75-0.83, adjusted for age and Charlson comorbidity index). An age-stratified analysis revealed that this sex difference was restricted to women aged 60 years or more (<60 years: RR = 0.97, 95% CI 0.87-1.07; ≥ 60 years: RR = 0.73, 95% CI 0.68-0.78). Overall 17.9% of all nephrectomies were performed as partial nephrectomies. The proportion of laparoscopically partial and total nephrectomies was 0.9% and 7.6% respectively. The most frequent type of nephrectomy was total open nephrectomy (71.6%) (Table 1).

**Table 1:** Characteristics of patients undergoing nephrectomy for the treatment of renal cancer in Germany, 2005-2006

	**All**	**Men**	**Women**
Hospitalizations	23,753	14,823	8,930
Lymph node (Ndl) and distant metastasis status			
Ndl -, distant metastasis -	89.4	89.0	90.0
Ndl +, distant metastasis -	1.7	1.7	1.7
Ndl +, distant metastasis +	1.7	1.9	1.4
Ndl -, distant metastasis +	7.2	7.4	6.9
Age (years) at hospitalization (%)			
< 40	2.9	2.8	3.1
40-49	8.0	8.6	6.8
50-59	17.7	19.6	14.5
60-69	33.4	35.0	30.9
70-79	30.2	28.5	33.1
≥ 80	7.8	5.5	11.5
Median age (P10-P90)	66 (49–78)	66 (48–77)	68 (50–80)
Length of hospital stay (days) Median (P10-P90)	11 (8–20)	11 (8–20)	12 (8–21)
Intrahospital mortality (%)	1.4	1.5	1.3
Charlson comorbidity index: Median (P10-P90)	1.0 (0–4)	1.0 (0–4)	0 (0–3)
Type and surgical approach (%)			
Partial (any)	17.9	19.4	15.3
Partial, open	17.0	18.4	14.7
Partial, laparoscopic	0.9	1.0	0.6
Total (any)	79.2	77.6	81.9
Total, open	71.6	70.1	74.3
Total laparoscopic	7.6	7.5	7.7
Unclear or implausible^1)^	2.9	3.0	2.7
Complications (%)			
Gastrointestinal tract	1.1	1.2	1.0
Respiratory system	2.7	2.8	2.5
Bleeding or acute posthaemorrhagic anemia	18.4	17.8	19.4
Bleeding or acute posthaemorrhagic anemia requiring blood transfusion	13.5	12.6	14.8
Urological system	2.3	2.6	1.7
Infections	1.9	2.0	1.8

Legend.

P10 and P90: 10^th^ and 90^th^ percentile; 1) Implausible combinations of codes like partial and simple nephrectomy or partial and radical nephrectomy; the corresponding codes for the type of nephrectomy, surgical approach, complications, and Charlson comorbidity index are presented in the supplementary online document.

Risks of gastrointestinal (mainly postoperative intestinal ileus) and respiratory (mainly acute respiratory or pulmonary insufficiency and pneumothorax) complications were 1.1% and 2.7% respectively. The risk of haemorrhage or an acute posthaemorraghic anemia was 18.4%. Haemorrhages were coded in 4.5% of all patients. The risk of haemorrhage or an acute posthaemorraghic anemia that required blood transfusion was 13.5%. The risk of urological complications was 2.3% and was mainly due to acute renal failure (in 521 out of 546 patients (95.4%)). The risk of infections was 1.9% (Table 1 & Additional file 1: Table S4). Whereas haemodialysis was performed among 1.7% (383/23,207) of patients without urological complications, this proportion was 27.7% (151/546) among patients with urological complications.

LOS was associated with the type of nephrectomy and complications after nephrectomy. In comparison to total open nephrectomy, total laparoscopic nephrectomy was associated with about −3.3 days shorter hospital stays after adjustment for age and comorbidities. Partial nephrectomy was only weakly associated with a shorter hospital stay (−0.8 days) (Additional file 1: Table S5). After adjustment for age, comorbidity, and type of surgery, all complications after nephrectomy were associated with a considerable prolongation of the estimated LOS. Infections (+12.8 days) and urological complications (+9.7 days) were associated with the longest prolongation of LOS (Additional file 1: Table S6).

The adjusted RR of gastrointestinal complications was lower for partial nephrectomy than total open nephrectomy (RR = 0.66, 95% CI 0.45-0.97). Furthermore, the adjusted RR of haemorrhage or acute posthaemorrhagic anemia was lower for total laparoscopic nephrectomy compared to total open nephrectomy (RR = 0.69, 95% CI 0.61-0.78). A restriction to those haemorrhages or acute posthaemorrahagic anemias that resulted in blood transfusion revealed an even lower risk for total laparoscopic nephrectomy compared to total open nephrectomy. The adjusted RR of urologic complications was increased for partial open nephrectomies compared to total open nephrectomy (RR = 1.45, 95% CI 1.18-1.78) (Table 2).

**Table 2:** Surgical approaches of nephrectomy, crude risks of in-hospital complications and adjusted relative risks of complications in Germany 2005-2006

	**Partial nephrectomy**	**Total nephrectomy**		**Adjusted relative risks of complications in comparison to total open nephrectomy**			
		**Open**	**Lap.**	**Partial**		**Total Lap.**	
**Subjects (N)**	**4,244**	**17,017**	**1,797**	**RR**	**95% CI**	**RR**	**95% CI**
**Complications (%)**							
Gastrointestinal	0.7	1.2	1.6	0.66	0.45-0.97	1.46	0.98-2.16
Respiratory	2.8	2.7	1.8	1.11	0.91-1.36	0.75	0.53-1.06
Bleeding or acute posthaemorrhage anemia	18.5	19.0	11.9	1.08	1.01-1.16	0.69	0.61-0.78
Bleeding or acute posthaemorrhage anemia requiring blood transfusion	12.1	14.4	7.2	0.96	0.88-1.05	0.57	0.48-0.67
Urologic	2.7	2.3	1.3	1.40	1.14-1.72	0.68	0.45-1.03
Infection	1.9	1.9	1.7	1.08	0.85-1.38	0.97	0.67-1.39

Legend.

Unclear nephrectomies (N = 695, 2.9%) not shown; Lap.: laparoscopic; relative risks are adjusted for age and Charlson comorbidity index; 95% CI: 95% confidence interval.

Charlson comorbidity index was positively associated with the LOS and risk of in-hospital death. Whereas patients with an index of zero had a median LOS of 11 days, patients with an index of seven or more had a median LOS of 19 days. The estimated age-adjusted increase of LOS per one unit increase of the Charlson comorbidity index was 1.3 days (95% CI 1.2-1.4 days). Per one unit increase of the index, the adjusted risk of in-hospital death increased by 53% (95% CI 47-59%) (Table 3). Overall 19.1%, 2.9%, 0.4%, and 0.07% of all nephrectomies were associated with one, two, three and four or more complications respectively. For subjects with no complications, the risk of in-hospital death was 0.6%. All complications were associated with increased adjusted RRs of in-hospital death. We observed the highest adjusted RR of in-hospital mortality for gastrointestinal (RR = 3.61) and urologic complications (RR = 3.62) (Table 4).

**Table 3:** Charlson comorbidity index, median length of hospital stay, observed in-hospital deaths and estimated adjusted relative risks of death in Germany, 2005-2006

							**Relative risk of in-hospital death**			
			**Length of hospital stay**		**Observed in-hospital deaths**		**Crude analysis**		**Adjusted analysis**	
**Charlson**	**N**	**%**	**Median**	**P10-P90**	**N**	**%**	**RR**	**95% CI**	**RR**	**95% CI**
0	11,687	49.2	11	8-16	38	0.3				
1	3,632	15.3	11	8-19	38	1.1	2.41	1.59-3.66	2.4	1.33-3.13
2	3,234	13.6	12	8-22	39	1.2	2.78	1.84-4.21	2.47	1.62-3.76
3	2,726	11.5	13	9-26	84	3.1	7.10	5.03-10.03	6.35	4.45-9.05
4	1,111	4.7	14	9-29	38	3.4	7.88	5.20-11.94	6.12	3.97-9.43
5	782	3.3	15	9-32	40	5.1	11.79	7.84-17.72	9.91	6.53-15.05
6	356	1.5	15	9-35	29	8.2	18.78	12.05-29.25	14.43	9.11-22.85
≥ 7	225	0.9	19	10-42	31	13.8	26.43	15.81-44.17	18.88	10.98-32.47
Per 1 unit increment							1.58	1.52-1.65	1.53	1.47-1.59

Legend.

Percentages are row percentages, that is, percent of deaths within levels of Charlson comorbidity indices; P10 and P90: 10^th^ and 90^th^ percentile; RR: relative risk; 95% CI: 95%% confidence interval; per 1 unit increment: estimated relative risk per 1 unit increase of the Charlson comorbidity index; IQR: interquartile range; adjustment for age (continuous).

**Table 4:** Association between complications after partial or total nephrectomy and risk of in-hospital death in Germany, 2005-2006

	**Frequency of complication**		**In-hospital deaths**		**Relative risk of in-hospital death**	
	**N**	**%**	**N**	**%**	**Adj. RR**	**95% CI**
No complication	18,415	77.5	108	0.6		
**Complication**						
Gastrointestinal	263	1.1	18	6.8	3.61	2.32-5.63
Respiratory	636	2.7	44	6.9	2.05	1.30-3.03
Urologic	546	2.3	102	18.7	3.62	2.62-5.00
Infection	449	1.9	18	4.0	2.45	1.47-4.07
Bleeding or acute posthaemorrhage anemia	4,371	18.4	188	4.3	2.11	1.73-2.57
Bleeding or acute posthaemorrhage anemia requiring blood transfusion	3,086	13.0	157	5.1	1.96	1.57-2.44

Legend.

Overall 23,753 hospitalizations included; for each complication, the reference group consists of hospitalizations without the corresponding complication; all relative risks are adjusted for age and Charlson comorbidity index.

Partial and laparoscopic nephrectomy was associated with a lower in-hospital mortality (adjusted RR = 0.39, 95% CI 0.25-0.62 and adjusted RR = 0.34, 95% CI 0.17-0.67, respectively). Patients with lymph node or distant metastases had higher mortality risks compared to patients with localized disease (adjusted RR = 1.63, 95% CI 1.12-2.38 and adjusted RR = 4.13, 95% CI 3.21-5.31, respectively).

## Discussion

Overall 17.9% of all nephrectomies were performed as partial nephrectomies. The age and comorbidity adjusted LOS was shorter for total laparoscopic nephrectomy than total open nephrectomy. The adjusted risk of bleeding or acute posthaemorraghic anemia was also lower for total laparoscopic nephrectomies compared to total open nephrectomies. Typical procedure-associated risks ranged between 1.1% and 2.7% with the exception of bleeding or acute posthaemorrhagic anemia, with 18.4%. All complications were associated with an increased LOS, especially infections and urologic complications. Furthermore, all complications were associated with increased risks of in-hospital deaths. Charlson comorbidity index was strongly associated with the LOS and risk of in-hospital death.

The proportion of partial nephrectomy for the treatment of renal cancer was 31% in Italy in 2007 [15] and 45.2% in the United States in 2006 [16]. A high proportion of partial nephrectomy probably reflects increased surgeon education and greater surgeon comfort with partial nephrectomy [16]. In our study, this proportion was considerably lower (17.9%) and may be due to the inclusion of renal cancer patients of any age and stage as compared to e.g. the USA that only included renal cancer patients aged 40–90 years with a maximum tumor diameter of 4 cm. Alternatively, the lack of incentives within the German hospital reimbursement may explain this finding as reimbursement is identical for open and laparoscopic nephrectomy. A potential disadvantage of partial nephrectomy is the risk of local recurrence estimated to be 3-6% [2] and its technical demand [17]. Similarly as in the US, we found that men had a higher probability undergoing partial nephrectomy than women [18].

Despite some standardization approaches of surgical complication reporting [5,6], definitions of surgical complications are frequently not reported and lack a consensus among urologists [6] resulting in risks of complications from 2% to 54% [19,20]. Bleeding can be distinguished into three forms: acute, postoperative, and delayed occurrence of bleeding [3]. Roos et al. used the percentage of patients who received a blood transfusion as a surrogate of perioperative bleeding and found that this proportion ranges between 6.8% and 18.0% [21]. If we used this definition, the risk of perioperative bleeding in Germany would be 19.1%. However, the coding of bleeding plus blood transfusion occurred in only 4.5%. This risk is in line with previous tertiary center studies [8,22]. When we also used codes that indicated the acute consequence of bleeding (acute postoperative haemorrhagic anemia), this risk increased to 18.4%. Kim et al. defined bleeding as the occurrence of haemorrhage or acute post haemorrhagic anemia and observed a rate of 8.5%, 5.3%, and 7.0% for open radical, laparoscopic radical, and open partial nephrectomy respectively [23]. These rates are still considerably lower than in our study. At least two reasons might explain the higher rates in Germany: first, in contrast to studies based on the National Inpatient Sample of the USA, we used all 100 ICD-10 codes to identify haemorrhage and acute post haemorrhagic anemia; second, recording of complications is triggered by reimbursement incentives [24] that may differ between the USA and Germany.

The estimated average volume of intraoperative blood loss during partial laparoscopic nephrectomy is 150–250 ccm [25]. Among 200 patients undergoing partial laparoscopic nephrectomy, overall 3.5% suffered from intraoperative haemorrhage. However, among these patients the mean blood loss was 1,425 ccm [26]. As others [21], we found that the median LOS was shorter and the risk of haemorrhage or acute postoperative haemorrhagic anemia that required blood transfusion was lower among patients undergoing total laparoscopic nephrectomy compared to total open nephrectomy. However, our study and many case series of nephrectomy may suffer from a selection effect also called confounding by indication: renal cancer treated by laparoscopic nephrectomy tends to have a lower stage at diagnosis [16]. The only staging information available in our data set was related to the presence of lymph node and distant metastases. Patients with lymph node or distant metastases were less likely to undergo laparoscopic nephrectomy than patients without metastases. Confounding by indication may also have influenced the association between type of nephrectomy (laparoscopic or partial, respectively) and in-hospital mortality in our analyses.

The adjusted relative risk of gastrointestinal complications (mainly postoperative intestinal ileus) was lower for partial nephrectomy compared to total open nephrectomy. Furthermore, the adjusted RR of postoperative intestinal ileus was higher among patients undergoing total laparoscopic nephrectomy as compared to total open nephrectomy. The higher adjusted RR of urologic complications (mainly renal failure) for partial nephrectomy may reflect a selection bias towards patients with either a single kidney or patients with chronic renal failure.

The adjusted increase of the LOS was largest for patients who acquired infections and renal failure. Renal failure after partial nephrectomy results from a combination of intraoperative ischemia and loss of functioning renal parenchyma. Chronic kidney disease is more common after radical nephrectomy than after partial nephrectomy, even in the presence of a normal contralateral kidney and a normal preoperative concentration of creatinine [27]. The degree of renal insufficiency is usually mild and can be treated with adequate transfusion and electrolyte management [28]. However, the considerably higher proportion of patients requiring haemodialysis associated with the occurrence of urologic complications (27.7%) compared to patients without this complication (1.7%) in our study indicates that acute urologic complications are serious complications.

Similar as in other studies, the perioperative mortality was very low [7,21,23]. The strong association between comorbidity and in-hospital mortality has several implications: first, renal cancer patients with comorbidities should be counseled about the mortality increase [29]; second, due to the ageing of Western industrialized populations and therefore increase of comorbidity among renal cancer patients, surgeons have to be aware of the comorbidity-associated risk of complications and in-hospital death; third, the quality of documentation and coding of comorbidities obviously has some validity as the expected well-known association between comorbidity and in-hospital mortality [19] could be replicated based on these nationwide hospitalization data.

Our study has several strengths. In contrast to the few previous population-based studies of the surgical management of renal cancer, we included all types of nephrectomies including laparoscopy and all stages of renal cancer including metastasized cancer. It presents virtually a nationwide, unbiased overview of all types of tumor nephrectomies undertaken in Germany and provides the most comprehensive and contemporary analysis of the use of nephrectomy for renal cancer treatment. Furthermore, we used all available comorbidity diagnoses (up to 100 ICD-10 codes) whereas previous studies used 3 [30], 6 [15], 15 [8], or up to 20 [7] diagnoses only to derive Charlson comorbidity index and complications.

However, there are inherent limitations of using administrative data in clinical research [24]. First, for the prediction of postoperative morbidity and mortality, several factors beyond the Charlson comorbidity index, type of nephrectomy and warm ischemia time play a role like tumor T-stage, location and grade, obesity, anesthetic techniques, surgeon experience and training, duration of surgery or anesthesia, availability of equipment and level of postoperative care [21,27]. Second, administrative hospitalization data lack information on severity (grade) of complications and therefore the Clavien classification of surgical complications [19] cannot be applied. We also could not estimate the reoperation risk for bleeding after nephrectomy. Case series analyses have shown reoperation rates for bleeding of 1.5% [22] and 2.2% [31]. Finally, our results are derived from 2005–2006 and management of kidney cancer may have changed in favor of laparoscopic nephrectomy.

## Conclusions

The nationwide analysis of tumor-related nephrectomies shows a low proportion of partial nephrectomies that may be due to the inclusion of all stages of renal cancers or a lack of reimbursement incentives. In the era of ageing populations, renal cancer patients with comorbidities should be counseled about their increased in-hospital mortality risk. Administrative hospitalization data can contribute to routine reporting of age- and comorbidity-adjusted complication and in-hospital mortality rates.

## Abbreviations

CCI: Charlson comorbidity index; DRG: Diagnosis related group; ICD-10-GM: International classification of diseases, 10th edition, German modification; LOS: Length of stay; OPS: Classification of operations and procedures; RR: Relative risk; 95% CI: 95% confidence interval.

## Competing interests

The authors declare that they have no competing interests.

## Authors’ contributions

AS wrote the study protocol, regulated the access to the DRG data, supervised the statistical analysis and SAS programming, programmed several parts of the presented analyses himself, interpreted the results and wrote the manuscript. CB: performed some of the statistical analyses; programmed some parts of the presented analyses himself, interpreted the results, and wrote the manuscript. Both authors read and approved the final manuscript.

## Pre-publication history

The pre-publication history for this paper can be accessed here:

http://www.biomedcentral.com/1471-2490/14/74/prepub

## Supplementary Material

Additional file 1: Table S1. Types of nephrectomy and OPS coding. **Table S2.** Surgical approach of nephrectomy and coding. **Table S3.** Charlson comorbidity and ICD-10 codes and weighting. **Table S4.** Complications associated with nephrectomy and ICD-10 codes. **Table S5.** Association between type of nephrectomy, surgical approach and estimated change in length of hospital stay in comparison to total open nephrectomy among patients with renal cancer in Germany, 2005–2006. **Table S6.** Association between the occurrence of complications after partial or total nephrectomy and length of in-hospital stay in Germany, 2005–2006. Click here for file
