# Supplementary material for: Renal surgery for kidney cancer in Germany 2005–2006: length of stay, risk of postoperative complications and in-hospital death
Source: BMC Urol. 2014 Sep 12;14:74. doi: 10.1186/1471-2490-14-74 (PMC4169703; doi:10.1186/1471-2490-14-74)
Supplement: Additional file 1: Table S1. — Types of nephrectomy and OPS coding. Table S2. Surgical approach of nephrectomy and coding. Table S3. Charlson comorbidity and ICD-10 codes and weighting. Table S4. Complications associated with nephrectomy and ICD-10 codes. Table S5. Association between type of nephrectomy, surgical approach and estimated change in length of hospital stay in comparison to total open nephrectomy among patients with renal cancer in Germany, 2005–2006. Table S6. Association between the occurrence of complications after partial or total nephrectomy and length of in-hospital stay in Germany, 2005–2006. [file 1471-2490-14-74-S1.doc]

**Supporting information file**

**Table S1 Types of nephrectomy and OPS coding**

| **Type of nephrectomy** | **OPS codes** |
| --- | --- |
| Partial nephrectomy | 5-553.00, 5-553.01, 5-553.02, 5-553.03, 5-553.0x, 5-553.10, 5-553.11, 5-53.12, 5-553.13, 5-553.1x, 5-553.x0, 5553.x1, 5-553.x2, 5-553.x3, 5-553.xx, 5-553.y |
| Total nephrectomy |  |
| Simple nephrectomy | 5-554.a0, 5-554.a1, 5-554.a2, 5-554.a3, 5-554.ax, 5-554.b0, 5-554.b1, 5-554.b2, 5-554.b3, 5-554.bx |
| Radical nephrectomy | 5-554.40, 5-554.41, 5-554.42, 5.554.43, 5-554.4x, 5-554.50, 5-554.51, 5-554.52, 5.554.53, 5-554.5x, 5-554.60, 5-554.61, 5-554.62, 5.554.63, 5-554.6x |
| Other or unspecified type of nephrectomy | 5-554.70, 5-554.71, 5-554.72, 5-554.73, 5-554.7x, 5-554.x0, 5-554.x1, 5-554.x2, 5-554.x3, 5-554.xx, 5-554.y |

**Table S2 Surgical approach of nephrectomy and coding**

| **Surgical approach** | **OPS codes** |
| --- | --- |
| Laparoscopic | 5-553.03, 5-553.13, 5-553.x3, 5-554.43, 5-554.53, 5-554.63, 5-554.a3, 5-554.b3, 5-554.x3, 5-554.73 |
| Open lumbal | 5-553.00, 5-553.10, 5-553.x0, 5-554.40, 5-554.50, 5-554.60, 5-554.a0, 5-554.b0, 5-554.x0, 5-554.70 |
| Open abdominal | 5-553.01, 5-553.11, 5-553.x1, 5-554.41, 5-554.51, 5-554.61, 5-554.a1, 5-554.b1, 5-554.x1, 5-554.71 |
| Open thoracoabdominal | 5-553.02, 5-553.12, 5-553.x2, 5-554.42, 5-554.52, 5-554.62, 5-554.a2, 5-554.b2, 5-554.x2, 5-554.72 |
| Other or unspecified | 5-553.0x, 5-553.1x, 5-553.xx, 5-553.y, 5-554.4x, 5-554.5x, 5-554.6x, 5-554.ax, 5-554.bx, 5-554.xx, 5-554.7x, 5-554.y |

**Table S3 Charlson comorbidity and ICD-10 codes and weighting**

| **Charlson Comorbidity** | **Factor** | **ICD-10-Code** |
| --- | --- | --- |
| Acute myocardial infarction | 1 | I21.x, I22.x, I25.2 |
| Congestive heart failure | 1 | I09.9, I11.0, I13.0, I13.2, I25.5, I42.0, I42.5–I42.9, I43.x, I50.x, P29.0 |
| Peripheral vascular disease | 1 | I70.x, I71.x, I73.1, I73.8, I73.9, I77.1, I79.0, I79.2, K55.1, K55.8, K55.9, Z95.8, Z95.9 |
| Cerebrovascular disease | 1 | G45.x, G46.x, H34.0, I60.x–I69.x |
| Dementia | 1 | F00.x–F03.x, F05.1, G30.x, G31.1 |
| Chronic pulmonary disease | 1 | I27.8, I27.9, J40.x–J47.x, J60.x–J67.x, J68.4, J70.1, J70.3 |
| Rheumatic disease | 1 | M05.x, M06.x, M31.5, M32.x–M34.x, M35.1, M35.3, M36.0 |
| Peptic ulcer disease | 1 | K25.x–K28.x |
| Mild liver disease | 1 | B18.x, K70.0–K70.3, K70.9, K71.3–K71.5, K71.7, K73.x, K74.x, K76.0, K76.2–K76.4, K76.8, K76.9, Z94.4 |
| Diabetes mellitus without chronic complications | 1 | E10.0x, E10.1x, E10.6x, E10.8x, E10.9x, E11.0x, E11.1x, E11.6x, E11.8x, E11.9x, E12.0x, E12.1x, E12.6x, E12.8x, E12.9x, E13.0x, E13.1x, E13.6x, E13.8x, E13.9x, E14.0x, E14.1x, E14.6x, E14.8x, E14.9x |
| Diabetes mellitus with chronic complications | 2 | E10.2x–E10.5x, E10.7x, E11.2x–E11.5x, E11.7x, E12.2x–E12.5x, E12.7x, E13.2x-E13.5x, E13.7x, E14.2x–E14.5x, E14.7x |
| Hemiplegia or paraplegia | 2 | G04.1, G11.4, G80.1, G80.2, G81.x, G82.x, G83.0–G83.4, G83.9 |
| Renal disease | 2 | I12.0, I13.1, N03.2–N03.7, N05.2–N05.7, N18.x, N19.x, N25.0, Z49.0–Z49.2, Z94.0, Z99.2 |
| Any malignancy, including lymphoma and leukemia, except malignant neoplasm of skin | 2 | C00.x–C26.x, C30.x–C34.x, C37.x–C41.x, C43.x, C45.x–C58.x, C60.x–C63.x, C66.x-C76.x, C81.x–C85.x, C88.x, C90.x–C97.x |
| Moderate or severe liver disease | 3 | I85.0, I85.9, I86.4, I98.2, K70.4, K71.1, K72.1, K72.9, K76.5, K76.6, K76.7 |
| Metastatic solid tumor | 3 | C77.x–C80.x |
| AIDS/HIV | 6 | B20.x–B22.x, B24.x |

**Table S4**

**Complications associated with nephrectomy and ICD-10 codes**

| **ICD-10 codes** | **Complication** | **N** | **%** |
| --- | --- | --- | --- |
|  | **Bleeding or acute posthaemorrhagic anemia^1^** | **4,238** | **17.8** |
| D62 | Acute posthaemorrhagic anemia | 3,888 | 16.4 |
| K66.1 | Haemoperitoneum | 21 | 0.1 |
| R58 | Haemorrhage, not elsewhere classified | 28 | 0.1 |
| T81.0 | Haemorrhage and haematoma complicating a procedure, not elsewhere classified | 1,037 | 4.4 |
|  | **Respiratory complications** | **851** | **3.6** |
| J93.0 | Spontaneous tension pneumothorax | 8 |  |
| J93.1 | Other spontaneous pneumothorax | 7 |  |
| J93.8 | Other pneumothorax | 17 | 0.1 |
| J93.9 | Pneumothorax, unspecified | 52 | 0.2 |
| J95.1 | Acute pulmonary insufficiency following thoracic surgery | 12 | 0.1 |
| J95.2 | Acute pulmonary insufficiency following nonthoracic surgery | 384 | 1.6 |
| J95.4 | Mendelson's syndrome |  |  |
| J95.80 | Iatrogenic pneumothorax | 160 | 0.7 |
| J95.88 | Other diseases of the respiratory tract after treatment | 21 | 0.1 |
| J96.0 | Acute respiratory failure | 240 | 1.0 |
|  | **Urological complications** | **546** | **2.3** |
| N17.0 | Acute renal failure with tubular necrosis | 13 | 0.1 |
| N17.1 | Acute renal failure with acute cortical necrosis |  |  |
| N17.2 | Acute renal failure with medullary necrosis |  |  |
| N17.8 | Other acute renal failure | 92 | 0.4 |
| N17.9 | Acute renal failure, unspecified | 197 | 0.8 |
| N99.0 | Postprocedural renal failure | 236 | 1.0 |
| N99.8 | Other postprocedural disorders of genitourinary system | 23 | 0.1 |
| N99.9 | Postprocedural disorder of genitourinary system, unspecified | 3 |  |
|  | **Infectious disease complications** | **491** | **2.1** |
| K65.0 | Acute peritonitis | 62 | 0.3 |
| K65.8 | Other peritonitis | 12 | 0.1 |
| K65.9 | Peritonitis, unspecified | 13 | 0.1 |
| T81.4 | Infection following a procedure, not elsewhere classified | 441 | 1.9 |
|  | **Gastrointestinal complications** | **263** | **1.1** |
| K91.3 | Postoperative intestinal obstruction (ileus) | 110 | 0.5 |
| K91.88 | Other postprocedural disorders of digestive system after treatment, not elsewhere classified | 39 | 0.2 |
| K91.9 | Postprocedural disorder of digestive system, unspecified |  |  |
| S36.0 | Injury of spleen | 78 | 0.3 |
| S36.1 | Injury of liver or gallbladder | 10 |  |
| S36.2 | Injury of pancreas | 8 |  |
| S36.3 | Injury of stomach |  |  |
| S36.4 | Injury of small intestine | 6 |  |
| S36.5 | Injury of colon | 5 |  |
| S36.6 | Injury of rectum | 0 |  |
| S36.7 | Injury of multiple intra-abdominal organs | 0 |  |
| S36.8 | Injury of other intra-abdominal organs | 15 |  |
| S36.9 | Injury of unspecified intra-abdominal organs | 0 |  |

**Legend**

Percentages below 0.1 are not presented; 1) To identify bleeding that required blood transfusion, we used OPS codes (8-800.0, 8-800.1, 8-800.7-) that indicated the application of blood transfusions (whole blood or packed red blood cells); cell counts of less than 3 precludes the reporting due to data confidentiality

**Table S5**

**Association between type of nephrectomy, surgical approach and estimated change in length of hospital stay in comparison to total open nephrectomy among patients with renal cancer in Germany, 2005-2006**

| **Type of nephrectomy and surgical approach** | **Crude** | |  | **Age-adjusted** | |  | **Age- & Charlson comorbidity index adjusted** | |
| --- | --- | --- | --- | --- | --- | --- | --- | --- |
|  | **Effect (days)** | **SE** |  | **Effect (days)** | **SE** |  | **Effect (days)** | **SE** |
| Total, open (reference group) |  |  |  |  |  |  |  |  |
| Total, laparaoscopic | -3.8 | 0.19 |  | -3.8 | 0.19 |  | -3.3 | 0.18 |
| Partial | -1.3 | 0.13 |  | -1.2 | 0.13 |  | -0.8 | 0.13 |
| Unclearly coded renal surgery | 0.9 | 0.30 |  | 0.9 | 0.30 |  | 0.9 | 0.29 |

**Legend**

Total nephrectomy includes simple and radical nephrectomy; SE: standard error; linear regression with adjustment by age (continuous) and Charlson comorbidity index (continuous); the observed median and mean number of days in hospital for patients undergoing total open nephrectomy was 12 and 13.9 days respectively.

**Table S6**

**Association between the occurrence of complications after partial or total nephrectomy and length of in-hospital stay in Germany, 2005-2006**

|  | **Length of stay** | |  | **Effect of complication on length of stay** | |
| --- | --- | --- | --- | --- | --- |
| **Complication** | **Median length of stay** | **P10-P90** |  | **Adjusted Effect (days)** | **SE** |
| None | 11 | 8-17 |  |  |  |
|  |  |  |  |  |  |
| **Complications** |  |  |  |  |  |
| Gastrointestinal | 14 | 9-35 |  | 6.0 | 0.46 |
| Respiratory | 14 | 9-34 |  | 5.2 | 0.30 |
| Urologic | 18 | 8-47 |  | 9.7 | 0.32 |
| Infection | 20 | 10-49 |  | 12.8 | 0.34 |
| Bleeding or acute posthaemorrhagic anemia | 14 | 9-29 |  | 3.9 | 0.12 |
| Bleeding or acute posthaemorrhagic anemia requiring blood transfusion | 15 | 10-31 |  | 4.7 | 0.14 |

**Legend**

For each complication, the reference group was hospitalizations without the corresponding complication; effects estimated by linear regression with adjustment for, age, Charlson comorbidity index (both continuous variables), and type of surgery (partial, total open, total laparoscopic, unclearly coded surgery)
